# The development of a novel ferric phytate compound for iron fortification of bouillons (part I)

**DOI:** 10.1038/s41598-020-61833-2

**Published:** 2020-03-24

**Authors:** Swarnim Gupta, Edwin Habeych, Nathalie Scheers, Sylvie Merinat, Brigitte Rey, Nicola Galaffu, Ann-Sofie Sandberg

**Affiliations:** 1 Chalmers University of Technology, Department of Biology and Biological Engineering, Food and Nutrition Science, 412 96 Gothenburg, Sweden; 2 Nestlé Research, Micronutrient group, Société des Produits Nestlé SA, CH-1000, Lausanne, 26 Switzerland

**Keywords:** Malnutrition, Nutrition

## Abstract

In a series of two studies, we report the development (this study) and evaluation (part II) of a novel ferric phytate compound designed as a condiment iron fortificant. Condiments are used as iron fortification vehicles to reduce the prevalence  of iron deficiency. The challenge for iron fortificants in e.g. a bouillon matrix is to avoid undesired sensory effects and to ensure a reasonable cost. We added phytic acid to chelate iron, and hydrolysed protein to counteract the inhibiting effect of phytic acid on iron bioaccessibility. We characterised four novel ferric phytate compounds, destabilised by hydrolysed plant protein or amino acids. Colour stability of fortified bouillons with ferric phytate compounds was superior to bouillons fortified with ferrous sulfate. The iron-phytate-hydrolysed corn protein compound (Fe-PA-HCP) resulted in highest cellular ferritin induction in Caco-2 cells, in both vegetable (36.1 ± 13.40 ng/mg protein) and chicken (73.9 ± 19.93 ng/mg protein) bouillon matrices as observed in the human Caco-2/HepG2 cell model. Iron uptake (as estimated by ferritin production) from the Fe-PA-HCP compound was about 55% (chicken bouillon) and 66% (vegetable bouillon) of the iron uptake from ferrous sulfate. Based on this study, the Fe-PA-HCP compound was chosen for further evaluation (part II).

## Introduction

### Iron fortification of foods to prevent iron deficiency

Iron deficiency continues to be a major public health problem of nutritional origin across the globe. Nearly 30% of the world’s population is anemic, half of which could be attributed to iron deficiency (IDA)^1,2^. The main reason for IDA in developing countries is the low intake of bioavailable iron from a monotonous plant-based diet. Iron is mainly absorbed from the diet by means of two different routes, either as non-heme iron from vegetables or other plant-based food sources, or as heme-bound iron. Heme-bound iron in meats or animal-based foods, is generally more bioavailable than non-heme iron, because of the protective environment of the iron provided by the heme molecule. Moreover, the heme molecule is not removed from the iron until the whole complex has crossed the intestinal membrane, unlike non-heme iron, which is transported into the intestine as free ions, susceptible to scavengers inhibiting the absorption. Potential solutions for preventing iron deficiency include dietary diversification and non-heme iron fortification of foods, the latter being generally accepted as the most cost-effective approach.

Iron fortification of common foods, like bouillons, has been proposed as a suitable strategy to reduce the prevalence of IDA. However, iron fortification of foods or beverages with relevant levels of iron constitutes a challenging process since the highest bioavailable forms of iron are chemically reactive and often cause detrimental side effects in the food matrix (e.g., colour change, metallic taste, and rancidity). By contrast, inert iron compounds are chemically stable in the food matrix but are low in bioavailability to humans. As a result, the choice of iron fortificant in the development of fortified food products is often a compromise between reasonable cost, stability in the formulation, and adequate bioavailability of the selected fortificant^3^. An example of an iron fortificant fulfilling the above requirements is Ferric pyrophosphate, a reference compound for fortification of condiments like bouillons. However, iron absorption from ferric pyrophosphate (FePP) tends to be low compared to ferrous sulfate (FeSO4), the reference compound for assessing iron bioavailability. Alternative iron compounds with high bioavailability and similar stability in products would therefore be valuable for fortification of condiments like bouillons^4,5^.

### Bioavailability of iron from mono ferric phytate

Phytic Acid (PA) is considered to be an inhibitor of iron absorption and is present abundantly in food grains, particularly in the cereal bran. It chelates iron and forms precipitates^6^, which are insoluble at intestinal pH and prevents iron and other metal ions to be bioavailable to monogastric animals, including humans, due to the lack of endogenous production of the enzyme phytase in the gastrointestinal tract^6,7^. The inhibitory effect of phytate on ferric iron was evident at equimolar ratio of phytate to iron^8,9^. However, mono ferric phytate, a major form of iron in wheat bran has also shown to be soluble and highly bioavailable in the rat^10^. In dogs and humans, mono ferric phytate was at least as well absorbed as ferric chloride^11,12^.

### The present study (Part I)

This proof of concept study is the first study out of two studies, in which we report the characterization of a novel iron compound Fe-PA-HCP (Ferric Phytate Hydrolysed Corn Proteins). In the second study (part II)^13^, we report the results of a human intervention trial of vegetable bouillons fortified with this compound. The idea of the present study was to investigate if protein hydrolysates (amino acids or peptides) along with iron (Fe) phytate (PA) complexes would be useful for iron fortification in regard to sensory attributes and absorption in gut epithelial cells. We used the Caco-2/HepG2 cell model for iron uptake studies^14^ based on the previous Caco-2 cell model, using ferritin as a marker for intestinal iron uptake. In the Caco-2/HepG2 cell model, hepatocytes are cultured in the basal medium of the Caco-2 cells, to allow for hepcidin regulation of iron uptake on the surface of the Caco-2 cell epithelium. Protein hydrolysates including certain amino acids and peptides of various length have already been shown to improve iron absorption by keeping iron soluble, reducing the ferric to ferrous iron, and promoting the transport across cell membranes, and offset the inhibitory effect of phytate^15^. However, the stability of the compounds including unacceptable colour changes in food  matrices is a concern^16,17^.

The Fe-PA-HCP compound was developed by screening for a stable formulation with ferric phytate and three basic polar amino acids (i.e., histidine, lysine, and arginine), cysteine and glutamine as neutral polar amino acid, and the amino acid glycine, which led to two stable formulations: Fe-PA-Histidine-Glutamine (Fe-PA-Hist-Gln) and Fe-PA-Histidine-Glycine (Fe-PA-Hist-Gly). The compounds were further optimized in term of cost effectiveness, whereby the amino acids were substituted with hydrolysed proteins from soy (Fe-PA-HSP) and corn (Fe-PA-HCP). Out of these four, the Fe-PA-HCP compound was chosen for iron absorption studies in the human intervention reported in the second paper of the studies. To be able to differentiate between absorbed iron from the Fe-PA-HCP compound and other ingested iron during the intervention, the naturally abundant ^56^Fe was exchanged for ^58^Fe and thus the same batch with ^58^Fe-PA-HCP was also used in the present study.

## Results

### Composition of the iron-phytate compounds

The composition of the four ferric phytate compounds; Fe-PA-His-Gln, Fe-PA-His-Gly, Fe-PA-HSP and ^58^Fe-PA-HCP are described in Table 1.

**Table 1:** Composition of the iron-phytate-amino acid/hydrolysed corn or soy protein compounds.

Compounds	Composition	% iron
Fe-PA-His-Gly	Iron, phytate, histidine, glycine in the molar ratio 1Fe:1PA:1His:2Gly	3.4 ± 0.2
Fe-PA-His-Gln	Iron, phytate, histidine, glutamine in the molar ratio 1Fe:1PA:1His:2Gln	3.4 ± 0.1
Fe-PA-HSP	Iron, phytate in molar ratio 1.5 Fe:1 PA with hydrolysed soy protein	5.1 ± 0.2
^58^Fe-PA-HCP	Iron, phytate in the molar ratio 1.4 Fe:1 PA with hydrolysed corn protein	5.8 ± 0.1

Iron estimation was done by Microwave Plasma-Atomic Emission Spectrophotometry (MP-AES). Values represent means ± SD, (n = 2).

### Morphology of the iron-phytate acid compounds

**Figure 1:**
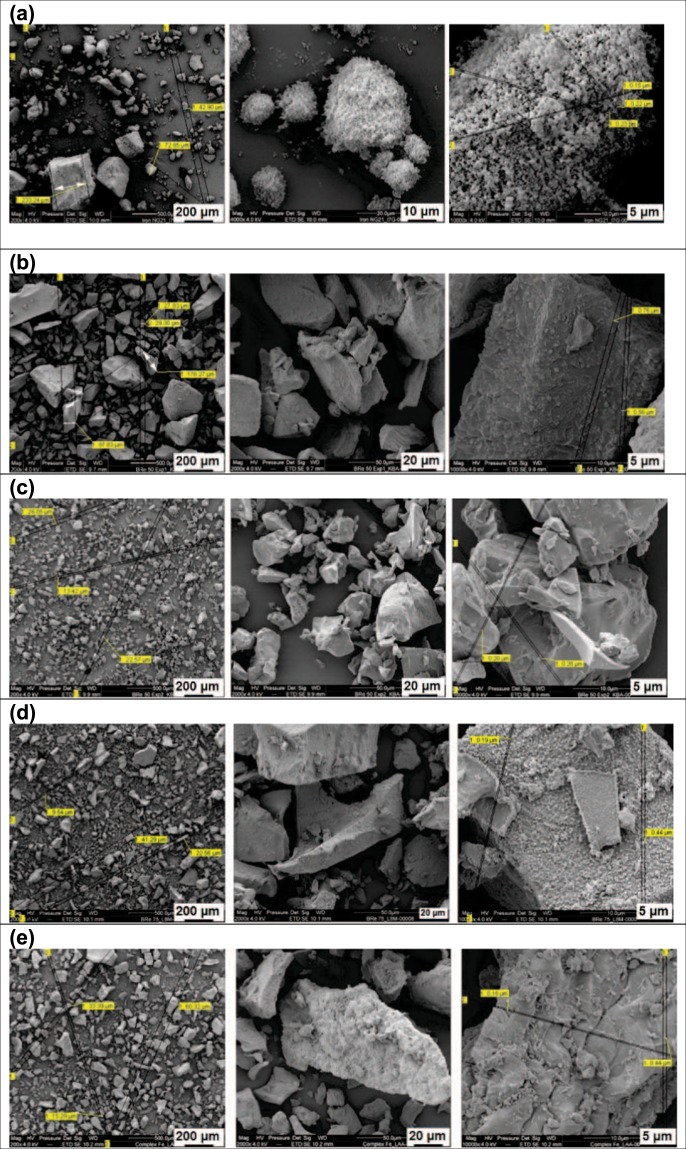
Low (left), medium (centre) and high (right) resolution Scanning Electron Microscopy micrographs of iron-phytate compounds. (**a**) Intermediate mono ferric phytate; (**b**) Fe-PA-His-Gly; (**c**) Fe-PA-His-Gln; (**d**) Fe-PA-HSP; (**e**) ^58^Fe-PA-HCP. Scale bar: 5–200 μm.

The effect of the addition of amino acids and hydrolysed protein on the morphology and particle size of the mono ferric phytate particles was investigated by Scanning Electron Microscopy (SEM), Fig. 1a–e. Figure 1a shows mono ferric phytate prior to the addition of the amino acids or hydrolysed proteins. The intermediate consisted of particulate material of average sizes of 200 μm formed by primary particles of approximately 0.2 μm. Furthermore, the addition of histidine and glycine to mono ferric phytate to obtain Fe-PA-His-Gly led to the formation of a mix of platelet and spherical particles of an average size of 50 μm (Fig. 1b). The morphology of Fe-PA-His-Gln is presented in Fig. 1c. The particles were 50 μm in average and had smooth surfaces with sharp edges. Moreover, the use of hydrolysed proteins either from soy (Fig. 1d) or corn (Fig. 1e) led to spherical and platelet-looking structures respectively, with the average size of 100 μm. Overall, the use of both amino acids and hydrolysed proteins had a profound impact on the morphology of mono ferric phytate particles by decreasing particle size.

### Improved colour stability of bouillons fortified with the iron-phytate compounds vs ferrous sulfate

**Figure 2:**
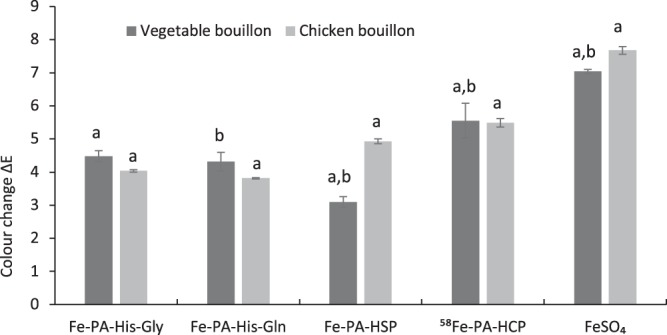
Overall colour change (∆E) of the bouillons in the presence of the iron fortificants based on CIE L*a*b* scale. Fortified bouillons were reconstituted with boiling water (95 °C) followed by cooking (60 °C, 30 min) prior to evaluation. Data are presented as means ± SD (n = 2–3). Two to three runs of the experiments conducted in triplicates resulted in 6–9 observations for each treatment. The letters a and b indicate significant differences between the treatments (p < 0.05) with respect to the particular matrix.

Comparative data of the overall colour difference, using the CIEL*a*b* scale, upon reconstitution of chicken and vegetable bouillon in boiling water followed by the addition of the ferric phytate compounds is presented in the Fig. 2. The results showed that the colour change (ΔE) of the bouillons was significantly smaller in samples fortified with ferric phytate compared to ferrous sulfate. The overall colour deviation for each compound was dependent on whether the bouillon matrix was composed of chicken or vegetables. The chicken bouillon fortified with the Fe-PA-amino acid compounds outperformed the Fe-PA- hydrolysed corn or soy protein compounds. In the vegetable bouillon matrix, the Fe-PA-HSP compound induced the significantly smallest change compared to all other compounds.

The total colour deviation (ΔE) of each sample from the reference (unfortified bouillon preparation) was based on the contribution of three parameters: (1) change of luminosity (ΔL > 0: sample brighter than reference, ΔL < 0: sample darker than reference); (2) change of red-green (Δa > 0: sample more red than reference, Δa < 0: sample more green than reference); (3) change of yellow-blue (Δb > 0: sample more yellow than reference, Δb < 0: sample more blue than reference). The comparison between these parameters is presented in Table 2. Changes in luminosity (ΔL) appeared to be the largest contributor to the overall colour change ΔE. In general, iron fortified samples became darker than the unfortified reference. The highest darkening was exhibited by ferrous sulfate. Δb turned to be the second largest contributor to the colour difference. In vegetable bouillons, the Fe-PA-Gly and Fe-PA-Gln compounds made the matrix more yellow than the reference while the other compounds exhibited a change towards the blue spectrum as observed for ferrous sulfate. The shift towards the blue was also observed for all iron compounds in chicken bouillon.

**Table 2:** Change in luminosity (ΔL) and colour gradient (Δa, Δb) in bouillon fortified with the iron compounds.

Matrix	Fortificant	ΔL	Δa	Δb
Vegetable bouillon	Fe-PA-His-Gly	−4.3 ± 0.2	0.9 ± 0.1	0.9 ± 0.2
Vegetable bouillon	Fe-PA-His-Gln	−3.9 ± 0.2	0.8 ± 0.0	1.5 ± 0.3
Vegetable bouillon	Fe-PA-HSP	−2.8 ± 0.2	0.8 ± 0.0	−2.0 ± 0.7
Vegetable bouillon	^58^Fe-PA-HCP	−5.1 ± 0.4	0.8 ± 0.0	−2.0 ± 0.7
Vegetable bouillon	FeSO_4_	−5.5 ± 0.1	3.8 ± 0.0	−2.3 ± 0.2
Chicken bouillon	Fe-PA-His-Gly	−3.3 ± 0.1	1.5 ± 0.0	−1.8 ± 0.2
Chicken bouillon	Fe-PA-His-Gln	−3.2 ± 0.1	1.5 ± 0.0	−1.5 ± 0.1
Chicken bouillon	Fe-PA-HSP	−4.0 ± 0.2	2.1 ± 0.0	−1.9 ± 0.2
Chicken bouillon	^58^Fe-PA-HCP	−1.9 ± 0.6	2.7 ± 0.0	−4.3 ± 0.2
Chicken bouillon	FeSO_4_	−5.7 ± 0.2	3.6 ± 0.0	−3.7 ± 0.1

Fortified bouillons were reconstituted with boiling water (95 °C) followed by simulated cooking (60 °C, 30 min) prior to evaluation. Values for ΔL > 0 indicate that samples are brighter and ΔL < 0 as darker than unfortified samples; Values for Δa > 0 indicate sample stronger red and Δa < 0 as stronger green than unfortified samples; Values for Δb > 0 indicate sample stronger yellow and Δb < 0 as stronger blue than unfortified. Data are presented as means ± SD (n = 2–3). Two to three runs of the experiment conducted in triplicates resulted in 6–9 observation for each treatment.

### Phytate negatively influenced iron bioaccessibility in the Caco-2/HepG2 cell model

**Figure 3:**
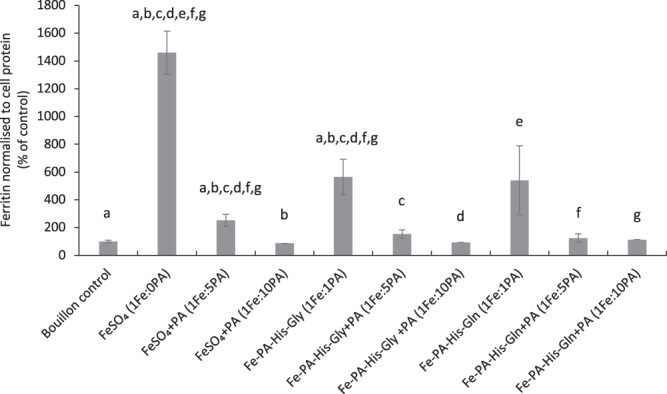
Bioaccessibility of iron from fortified chicken bouillon containing 8.9% dephytinized rice bran in the presence of phytate using simulated gastrointestinal digestion coupled to the Caco-2/HepG2 co-culture cell model. Ferritin levels in Caco-2 cells (normalized to cellular protein) in response to supernatants from digested samples containing different iron compounds with or without exogenously added phytate at an iron to phytate ratio of 1Fe:5PA and 1Fe: 10 PA are presented as percentages of ferritin in cells exposed to digested unfortified chicken bouillon. The bars represent means ± SD (n = 3). The letters (**a**–**g**) indicate significant differences between the treatments at p < 0.05.

The Fe-PA-His-Gly and Fe-PA-His-Gln compounds (compounds with higher ratio of PA:Fe (1:1) than in the Fe-PA-hydrolysed corn and soy protein compounds; PA:Fe of 1:1.4–1.5) and the iron reference salt Ferrous sulfate, diluted in chicken bouillon, were subjected to simulated gastrointestinal digestion in the presence and absence of added phytate at two molar ratios (1Fe:5PA and 1Fe:10 PA) followed by the incubation of Caco-2 cells with the digests in the Caco-2/HepG2 co-culture model. Induction of ferritin in Caco-2 cells, showed overall a decreasing trend in the presence of increasing phytate concentration (Fig. 3). The bioaccessibility of iron from the Fe-PA-amino acid compound was about 40% of the bioaccessibility of ferrous sulfate at a ratio of 1PA: 1Fe. A steep decrease in iron bioaccessibility of about 3.7 to 4.3 times was observed when the molar ratio of phytate to iron increased from 1PA:1Fe to 5PA: 1Fe in response to the compounds Fe-PA-His-Gly and Fe-PA-His-Gln. This decrease was maintained (4.8–6 times compared to 1Fe:1PA) if the ratio was further increased to 1Fe: 10 PA.

### Cellular bioaccessibility of iron from fortified vegetable bouillon

**Figure 4:**
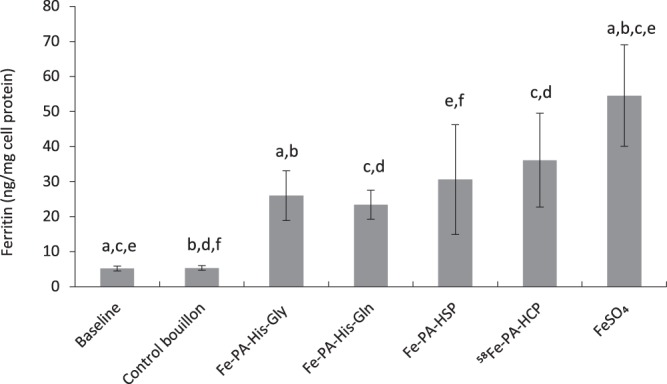
Bioaccessibility of iron from fortified vegetable bouillon in a simulated gastrointestinal digestion coupled to the Caco-2/HepG2 cell model. In these experiments, the whole digests without centrifugation was added to the cells to more closely mimic the reality. Two controls are presented; the baseline, which is the ferritin levels in untreated cells and the control bouillon, which is the ferritin levels in cells treated with unfortified vegetable bouillon digests. The cellular iron exposure was 100 µM of iron from the digested fortified bouillon samples. Experiments were conducted at least in quadruplicate and replicated once to generate eight or more observations per treatment. Data are presented as means ± SD (n = 2). The letters (**a**–**f**) indicate significant differences between the treatments at p < 0.05.

Iron fortified bouillon induced ferritin production in the Caco-2 cells in the Caco-2/HepG2 cell model, while the unfortified bouillon resulted in minimal synthesis similar to that of baseline (with no digests). Cellular (Caco-2) ferritin levels, in the presence of the bouillon fortified with the compounds were 23.4 ± 4.1 ng/mg protein (for Fe-PA-His-Gln), 26.0 ± 7.1 ng/mg protein (for Fe-PA-His-Gly), 30.6 ± 15.7 ng/mg protein (for Fe-PA-HSP), 36.1 ± 13.4 ng/mg protein (for ^58^Fe-PA-HCP) as compared to a ferritin induction of 56.5 ± 4.1 ng/mg protein with the ferrous sulfate control. This ferritin induction from the compounds corresponds to 42–66% of bioaccessible iron compared to the ferrous sulfate control (Fig. 4). The iron compound ^58^Fe-PA-HCP induced significantly higher ferritin production compared to Fe-PA-His-Gly and Fe-PA-His-Gln (p < 0.5) as could be expected due to the lower PA in the ^58^Fe-PA-HCP compound.

### Cellular bioaccessibility of iron from fortified chicken bouillon

**Figure 5:**
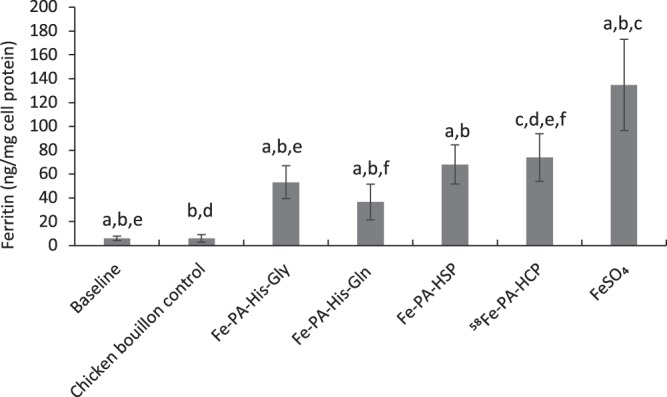
Bioaccessibility of iron from fortified chicken bouillon after simulated gastrointestinal digestion coupled to the Caco-2/HepG2 cell model. In these experiments, the whole digests without centrifugation was added to the cells to more closely mimic the reality. The cellular exposure was 100 µM of iron from the digested fortified bouillon samples. Two controls are presented; the baseline, which is the ferritin levels in untreated cells and the control, which is the ferritin levels in cells treated with unfortified chicken bouillon digests. The experiments were conducted in quadruplicates and replicated three times to generate 12 observations per treatment. Data are presented as means ± SD (n = 3). The letters (**a**–**f**) indicate significant differences between the treatments at p < 0.05.

The trend of the results was similar to that observed with the fortified vegetable bouillon. However, the magnitude of the ferritin production was slightly lower with the chicken bouillon matrix, as compared to the ferrous sulfate controls. The four compounds resulted in cellular ferritin synthesis of 36.6 ± 14.9 (Fe-PA-His-Gln), 53.3 ± 13.9 (Fe-PA-His-Gly), 68.0 ± 16.4 (Fe-PA-HSP), and 73.9 ± 19.9 ng/mg protein (^58^Fe-PA-HCP) that was about 27–54% of the ferrous sulfate reference (134.8 ± 14.92 ng/mg protein) in the chicken bouillon matrix. Also, the two amino acid compounds (Fe-PA-His-Gln and Fe-PA-His-Gly) with higher PA:Fe ratio had significantly less bioaccessibility compared to the hydrolysed protein-based Fe-PA-HSP and ^58^Fe-PA-HCP compounds (Fig. 5).

## Discussion

The major challenges for iron fortification of matrices such as bouillons are to choose an appropriate iron compound with high bioavailability as well as minimal impact on taste and stability of the product. Today, the iron compound of choice in bouillons is ferric pyrophosphate. However, iron from ferric pyrophosphate is low in bioavailability. Absorption rates of 13–39% in comparison to ferrous sulfate have been reported in human studies^18,19^. Although, ferrous sulfate is highly bioavailable, it is not suitable as a fortificant in food since it causes unacceptable sensory changes such as metallic taste and discoloration^20^, it was observed in the present study that ferrous sulfate caused yellow-coloured flocculation at the surface of the bouillons, which was not observable in the bouillons containing iron-phytate compounds. However, all fortified samples presented a shift towards the red, which may be expected due to the presence of phenolic compounds in the bouillon recipes. Bouillons are mostly not consumed as such but require addition of other foods such as vegetables or meats, which will also affect the color and palatability. Therefore, the future perspective of the present studies is that we need to conduct sensory evaluation studies of fortified bouillons in the context of local dishes

This is indeed the challenge; a compound in which the ligand has high affinity for iron, will unlikely be very bioavailable, but the stability of the product will be high. The common intestinal uptake route for iron is via the iron transporter DMT-1, which transports iron as ferrous iron. It is basically a competition between the transporter and the iron ligand. The optimal iron compound has an iron ligand, high enough in affinity for iron, to not react with the bouillon matrix, but low enough to release iron (ferric or ferrous) at the site of absorption. Ferric iron must first be reduced to ferrous iron, by means of the reductase Dcytb or by reducing agents in the gastrointestinal tract such as ascorbic acid^21^. In the light of this reasoning, it is perceivable that the colour stability of the bouillon matrix remains unaltered in the presence of phytate, a strong iron chelator, compared to ferrous sulfate in which the sulfate ions are free in solution, rendering the ferrous iron free to react with whatever that can donate an electron, and thereby causing colour and taste changes of the bouillons. Phytate (phytic acid is the protonated form), which is the form present at pH 7 is known to suppress adverse iron-catalysed reactions in food matrices, such as oxidation^22^. Its high affinity for iron also makes phytate a well-known inhibitor of iron absorption. Zwitterions such as amino acids and hydrolysed proteins (a mix of amino acids and small peptides) have a generally good solubility in water and were included to add charges and partially break the P-O-Fe bonds of the neutrally charged polymeric mono ferric phytate and thereby destabilising the coordinate-covalent bond between iron and phytate. In the present study, we found that at an iron to phytate ratio of 1.4–1.5 Fe: 1PA, in the presence of HCP or SHP, the bioaccessibility of iron to human intestinal cells (Caco-2) was about 50–66% of the bioaccessibility of ferrous sulfate, with remaining color stability of the bouillons in the presence of these iron compounds. We therefore conclude that the Fe-PA-HCP and Fe-PA-HSP compounds are promising vehicles for iron fortification of condiments like bouillons. Out of these two, the Fe-PA-HCP compound has two advantages; it is less costly to produce, and the corn proteins are not associated with allergenic properties, in contrast to the soy proteins. The prevalence of soy allergy in the population has been reported as 0.3–1.1%. To our knowledge, there are no valid reports on allergy (with IgE production) to corn proteins.

## Materials and methods

Hydrochloric acid (1 N) was purchased from Merck KGaA (Darmstadt, Germany). Food grade ammonium hydroxide (29% NH_3_) was acquired from Spectrum Chemicals MFG Corp (New Jersey, USA) and phytic acid 50% solution in water (w/v) was from Tongxiang Xinyang Food Additives Co., Ltd. (Tongxiang, China). Food grade ferrous sulfate heptahydrate was sourced from Dr Paul Lohmann GmbH KG (Emmerthal, Germany). Isotope-labelled [^58^Fe_2_]-(SO_4_)_3_ anhydrous (^58^Ferric sulfate as precursor of [^58^Fe]-PAHCP) was prepared in powder form by Dr. Paul Lohmann GmbH KG (Emmerthal, Germany) from isotope- enriched elemental iron ([^58^Fe] metal: 96.2%; ISOFLEX, San Francisco, USA). Food grade histidine was bought from Kiowa Inc. (Shangai, China), Glutamine from BioKiowa Inc. (Montreal, Canada), and glycine from Evonik Industries (Ham, France). Chemically hydrolysed protein from soy was purchased from Sigma-Aldrich (Saint Louis, USA) and from corn from Exter B.V. (Zaandam, Netherlands). Chicken and vegetable bouillons used for the colour stability and cell bioavailability studies were bought in a local store in Lausanne (Switzerland). Details on the materials used for cellular bioaccessibility are provided under the respective section.

### Production of the iron-phytate-amino acids/hydrolysed corn or soy protein compounds

Depending on the final amount of product, in a 0.2 L or 2 L double jacked reactor equipped with mechanical stirrer, FeSO_4_ (23% iron content) dissolved in Milli-Q water (18.2 MΩ), was added dropwise to a solution of PA (50% in weight) at pH 1.7 under agitation (500 rpm) to generate mono ferric phytate as a white precipitate, while the temperature was kept at −2 °C in circulating water bath with a flow rate of 15 mL/min. After, the amino acids or the hydrolysed proteins were dissolved in a minimum amount of Milli-Q water and added to the reactor. Subsequently, the solution was neutralized with 29% ammonium hydroxide to a final pH of 6.5 ± 0.5. Then, the resulting mix was stirred overnight (16 h) to achieve a clear solution that was pasteurized at 65 °C for 30 min, freeze dried (Telstar, LyoBeta 15, Terrassa, Spain), milled (Retsch, Ultra Centrifugal Mill ZM 200, Haan, Germany), and sieved (≤2 mm mesh) yielding a light yellow/white powder.

### Production of [^58^Fe]-PAHCP

The isotope enrichment of the starting materials; ^58^Fe metal powder and ^58^Fe_2_(SO_4_)_3_ was 96.2% and 95.27% respectively. The production of [^58^Fe]-PAHCP was conducted in a temperature-controlled reactor with mechanical stirring. Briefly, phytic acid solution (50%; 6.2 g) was diluted in water (18.2 MΩ; 50 mL) in the reactor. ^58^Fe_2_(SO_4_)_3_ (760 mg) was dissolved in water (18.2 MΩ; 50 mL) and added dropwise to the phytic acid solution (−2 °C, pH 1.7, 500 rpm agitation), mono ferric phytate was generated as a white precipitate. Furthermore, a solution of water (18.2 MΩ; 30 mL) and hydrolysed corn proteins (2.2 g) was added to the reactor. After that, the mixture was neutralized with ammonium hydroxide (29%) to a final pH of 6.5 ± 0.5 and then left in the reactor for 16 hours (with stirring). The preparation was pasteurized (65 °C; 30 min), freeze-dried, milled and sieved (≤2 mm mesh) resulting in a light yellow/white powder.

### Chicken and vegetable bouillons

Maggi, chicken Bouillon Powder (Nestlé, Switzerland) contained the following ingredients: Iodized Salt, Yeast Extracts, Natural X Flavourings (Eggs), White Sugar, Chicken Meat (antioxidants), Chicken Meat, Chicken Fat (Rosemary Extract), Onion, Onion, Vegetable Extracts (Leek Extract, Carrot Extract, Onion Extract, Garlic Extract), Spices (Curcumin, Nutmeg), Herbs (Parsley, Bay Leaf), Caramelized Sugar, Natural Celery Flavour. The bouillons were reconstituted in hot water at 12 g/500 mL (60 °C, 30 min). The nutritional content per 100 mL bouillon was 0.1 g fat, 0.3 g carbohydrates, 0 g dietary fiber, 0.2 g protein, 0.7 g salt.

Maggi, vegetable Bouillon Powder (Nestlé, Switzerland) contained: Iodized Salt, Vegetables (Onion, Carrot, Spinach, Celery), Yeast Extracts, White Sugar, Potato Starch, Vegetable Extracts (Carrot Extract, Leek Extract, Onion Extract, Garlic Extract), Sunflower Seed Oil, Natural flavourings, Spices (Curcumin, Paprika, Nutmeg), Chervil, Caramelized Sugar, Natural Celery Flavour. The vegetable bouillons were reconstituted in hot water at 13.1 g/500 mL (60 °C, 30 min). The nutritional content per 100 mL vegetable bouillon was 0 g fat, 0.4 g carbohydrates, 0.1 g dietary fiber, 0.2 g protein, 0.57 g salt.

### Scanning electron microscopy (SEM)

The samples were examined on a SEM FEI Quanta FEG 200 microscope (Thermo-Fisher, Eindhoven, Netherlands). After mechanical homogenisation, a small portion of the powdered sample was taken and dispersed on an aluminum stub covered with a conductive carbon tape. The stub was shaken to allow a good spread of powder. Excess powder was mechanically removed by the strong accelerations induced by tapping. The samples were then coated with a 10 nm gold layer using SCD 500 sputter coater (LEICA, Wetzlar, Germany) before being inserted in the electron microscope’s sample holder. The microscope’s acceleration voltage was set to 20 kV, spot size of 3 and a working distance of 18 mm.

### Colour measurement of fortified bouillons

Fortified chicken and vegetable bouillon samples were prepared in 1 L glass bottles by weighting 13.1 g of bouillon with different amounts of iron fortificants to obtain a fortification level of 84 mg of iron per 100 g of bouillon. This amount of iron corresponded to approximately 15% of the nutrient reference values for an adult^17^. Subsequently, 500 mL of boiling water was added to the dry mix and the resulting reconstituted bouillon was kept in a water-bath (Model 10128-128, VWR International, UK) at 60 °C for 30 minutes.

Colour analysis was carried using Standard CIE L*a*b* scale as described previously^23^. The measurements of a* (the amount of red and green), b* (the amount of yellow and blue), L* (the amount of luminosity from black to white) were recorded in triplicate from three independent samples obtained for each treatment using a reflectance spectrometer (ColourEye CE 7000 A, Gretagmacbeth, X-rite Europe AG provided with the IQC V7.0 software) set to a D65 illuminant and a 10° observer. Total colour deviation (ΔE) of each sample was calculated according to equation (1).

1 $$\Delta E=\sqrt{{({{\rm{L}}}_{{\rm{control}}}^{\ast }-{{\rm{L}}}_{{\rm{sample}}}^{\ast })}^{2}+{({{\rm{a}}}_{{\rm{control}}}^{\ast }-{{\rm{a}}}_{{\rm{sample}}}^{\ast })}^{2}+{({{\rm{b}}}_{{\rm{control}}}^{\ast }-{{\rm{b}}}_{{\rm{sample}}}^{\ast })}^{2}}$$

(Eq. 1) here ΔE refers to a measure of the overall colour change in the sample. ${{\rm{L}}}_{{\rm{sample}}}^{\ast }\,{{\rm{a}}}_{{\rm{sample}}}^{\ast }\,{{\rm{b}}}_{{\rm{sample}}}^{\ast }$ corresponds to the average measurement for the fortified and ${{\rm{L}}}_{{\rm{control}}}^{\ast }\,{{\rm{a}}}_{{\rm{control}}}^{\ast }\,{{\rm{b}}}_{{\rm{control}}}^{\ast }$ for the unfortified bouillon on CIE L*a*b* scale. This method has previously been used for colour stability estimation of food products^24^ and to understand the complexation ability of iron with various organic compounds in aqueous solution^25^.

## Bioaccessibility studies

### Experimental design

Simulated gastrointestinal digestion of samples coupled with iron uptake as estimated with ferritin formation, in Caco-2 cells using the Caco-2/HepG2 co-culture model^14^ was used to study the effect of phytate addition on iron bioaccessibility from iron compounds in chicken bouillon (with 8.9% dephytinized rice bran). The bouillons were fortified with two novel iron-phytate-amino acid complexes (Fe-PA-His-Gly and Fe-PA-His-Glu. In addition, the bioaccessibility of iron from four different Fe-PA-amino acid/hydrolysed corn or soy proteins in either a vegetable or a chicken bouillon matrix was investigated. Every experiment had control treatments; digested bouillon containing FeSO_4_, digested unfortified bouillon and no added digests (baseline control)

### Simulated gastrointestinal digestion of fortified bouillons

Unless specified, all reagents were obtained from Sigma-Aldrich, St. Louis, MO, USA. Briefly, 1 g of fortified bouillon (containing 1.2 mg of iron from the complex/FeSO_4_) was reconstituted in 18.2MΩ water (10 mL) and the pH lowered to 2.0 with 5 N HCl. This was incubated for 1 h (at 37 °C and 140 rpm) with 0.3 mL of pepsin (porcine gastric mucosa, Sigma-Aldrich; 2000 U/mL of HCl). The pH was then raised to 7 using 1 M NaHCO_3_ and incubated for 1 h (at 37 °C and 140 rpm) with 1.7 mL of bile-pancreatin solution (8.5 mg/mL bile and 1.4 mg/mL pancreatin in 0.1 M NaHCO_3_). After this, the final volume was adjusted to 15 mL using 18.2MΩ water. This protocol was based on Glahn *et al*.^26^.

For the experiment 1, where the effect of PA on iron bioaccessibility from the compounds were studied, the preparation of the sample and digestion procedure was followed with minor modifications as (a) the samples with and without PA (sodium salt; A and Z Additive Food Co. Ltd., Hangzhou, China) were incubated with the pepsin solution containing 178,200 U of pepsin per mL of 0.1 N HCL (b) samples were incubated for 0.5 h with 1.7 mL of pancreatin 4 g/L of 0.1 NaHCO_3_ without bile salt (c) the digested samples were centrifuged for 0.5 h at 500 g and supernatant transferred to a fresh vial. An aliquot (2 mL) of the supernatant was analysed for iron content using MP-AES 4200 (Agilent, Switzerland) method.

### Cell culture and treatment

The cell lines Caco-2 and HepG2 were obtained from the American Type Culture Collection (Rockville, MD, USA) and used at a passage 35–43 and 85–93, respectively. Unless, otherwise stated media, supplements and other reagents for cell culture were obtained from Gibco (Life Technologies, Paisley, UK).

The cells were grown in modified Eagle’s medium (MEM) with Earle’s salt supplemented with fetal bovine serum (FBS) (10%) and glutamine (1%) and an antibacterial, antifungal and anti-mycoplasma agent (0.2%; Normocin, Invivogen, Toulouse, France). The cells were grown at 37 °C in constant humidity (95%) and CO_2_ (5%). The medium was replaced every second day and cell were passaged at 70–80% confluency using trypsin-EDTA (0.5%, Life technologies). For conducting the experiments, Caco-2 were seeded in 12-well plates on trans-well polyester inserts (0.4 µm, 1,12 cm^2^) at a density of 60,000 cells per insert (#3460, Corning, Kennebunk, ME, US). Simultaneously, HepG2 were seeded in 12-well plates (3.8 cm^2^), at 200,000 cells/well (#3336, Corning, Kennebunk, ME, USA) according to the previous protocol^14^. Thirteen days post-seeding, inserts containing Caco-2 cells were transferred to the wells with HepG2 cells and allowed to equilibrate for 24 h with fresh media (supplemented with 1% FBS).

On day 14^th^, combined Caco-2/HepG2 cells were treated in triplicates/quadruplets at the apical (Caco-2 cell) surface with 0.5 mL of media (with 1% FBS) containing whole digests/supernatants (digests diluted in the existing media (1:1) to get a final iron concentration of 100 µM; or without digests (baseline control) and incubated for 2 h. At the end of the incubation, the apical media was aspirated, and the Caco-2 cells were washed with DPBS (Dulbecco’s Phosphate Buffer Saline without calcium and magnesium; GE Healthcare Life Sciences, Logan, UT, USA). The cells (Caco-2 and HepG2) were incubated for another 22 h after addition of fresh medium (with 1% FBS) to allow time for ferritin synthesis in the Caco-2 cells. The cells (Caco-2 and HepG2) were then washed with DPBS twice and lysed in ice-cold RIPA buffer (100 µL; Art. Cat. No. R0278, Sigma Aldrich) with added phosphatase and protease inhibitors (Pierce, Art. No. 88669; Rockford, IL, USA).

### Estimation of ferritin and total protein in Caco-2 cell lysates

Cellular ferritin formation was measured by a Sandwich ELISA kit (DRG, Gmbh, Germany) according to the manufacturer’s instructions. Total protein content was estimated with the BCA protein kit (Pierce, Rockford, IL, USA) method adopted to 96-well plate format. The final ferritin values reported for each cell lysate was normalized to its total protein content. For the experiment in which supernatants were used, the values were further normalized to the iron content of the supernatants to account for the differing iron concentrations of the treatments.

### Iron analysis

Iron contents of samples were determined by atomic emission spectrometry, using a MP-AES 4200 (Agilent, Switzerland). For MP-AES analysis samples (approx. 100–400 mg) were mineralized in duplicate in a Microwave Digestion System (Mars 6, CEM, USA) using Xpress microwave bombs with 4 mL of 70% HNO_3_ supra pure quality (Sigma-Aldrich, St. Louis, MO, USA 1 mL of 30% H_2_O_2_ (Merck KGaA, (Darmstadt, Germany). Mineral solutions were then transferred to 50 mL Falcon tubes and the volume was adjusted to 20 mL with Milli-Q water. Iron content was measured using external calibration with multi element standards at the wavelength 371 nm. Accuracy of the analysis was checked by analyzing the standard reference materials (SRM 3233, Typical Diet; NIST, MD, USA). Iron content was expressed as % w/w (compounds) or ppm (supernatant).

### Statistical analysis

The information on number of runs or observations for each experiment are provided along with the figures under the result section. The signals of the samples were first related to respective control for each run and then these values were collated for significance testing. Two-tailed Welch’s ‘t’ test was used because of heterogeneity in the variances, and differences were significant at p < 0.05. All the statistical analyses were carried out in IBM® SPSS® statistic version 24 (Chicago, IL, USA).
